# Survival benefits of propofol-based versus inhalational anesthesia in non-metastatic breast cancer patients: a comprehensive meta-analysis

**DOI:** 10.1038/s41598-024-67291-4

**Published:** 2024-07-16

**Authors:** Yingjun Zhang, Ping Yu, Lei Bian, Wanwei Huang, Na Li, Feng Ye

**Affiliations:** 1 Department of Anesthesiology, State Key Laboratory of Oncology in South China, Guangdong Provincial Clinical Research Center for Cance, Sun Yat-Sen University Cancer Center, Guangzhou, People’s Republic of China; 2 Department of Breast Oncology, State Key Laboratory of Oncology in South China, Guangdong Provincial Clinical Research Center for Cancer, Sun Yat-Sen University Cancer Center, Guangzhou, People’s Republic of China

**Keywords:** Inhalational anesthesia, Propofol-based anesthesia, Breast cancer, Survival, Comprehensive meta-analysis, Cancer, Oncology

## Abstract

Whether the anesthesia technique, inhalational general anesthesia (IGA) or propofol-based anesthesia (PBA), influences the long-term survival of non-metastatic breast cancer (eBC) remain unclear and controversial. We carried out a literature search on 16thJuly, 2022 for studies comparing IGA and PBA in eBC undergoing standard surgery, according to PRISMA 2020. The major endpoint in our study was overall survival (OS). Seventeen studies including four randomized clinical trials and thirteen retrospective cohort studies were included in the meta-analysis. Ten studies provided data for crude OS in unweighted eBC patients (imbalance in baseline characteristics). The summarized estimate HRs of the PBA group versus the IGA group (ten studies, N = 127,774, IGA group: 92,592, PBA group: 35,182.) was 0.83 (95%CI: 0.78–0.89). Compared with IGA, PBA was associated with both better 1-year OS (two studies, N = 104,083, IGA group: 84,074, PBA group: 20,009. Pooled HR = 0.80, 0.73–0.89) and 5-year OS (six studies, N = 121,580, IGA group: 89,472, PBA group: 32,108. HR = 0.80, 0.74–0.87). Ten studies applied PSM method to balance the baseline characteristics. In these weighted patients, PBA still showed a better OS (ten studies, N = 105,459, IGA group: 79,095, PBA group: 26,364. HR = 0.93, 0.87–1.00), a better 1-year OS (two studies, N = 83,007, IGA group: 67,609, PBA group: 15,398. HR = 0.88, 0.78–0.98) and a trend towards a better 5-year OS (nine studies, N = 121,580, IGA group: 76,797, PBA group: 24,066. HR = 0.95, 0.88–1.03). Loco-regional recurrence-free survival (LRRFS) was also better in PBA group (HR = 0.73, 0.61–0.86). The present study is the first comprehensive meta-analysis to demonstrate that propofol-based anesthesia could significantly improve OS and LRRFS in non-metastatic breast cancer patients, compared with inhalational anesthesia.

## Introduction

Breast cancer (BC) is the most common malignancy and is the leading cause of cancer mortality in women^1^. Mortality of early-stage/non-metastatic breast cancer (eBC) is usually attributable to cancer relapse including loco-regional recurrence and distant organ metastasis despite standard surgical resection, either mastectomy or breast conserving surgery. Surgery is still the primary and most effective treatment for eBC, but residual disease in the form of scattered micro-metastases and tumour cells is usually unavoidable. A series of effective treatments have been developed to improve the prognosis, including chemotherapy, endocrine therapy, target therapy, radiotherapy and immune therapy, etc.^2,3^. However, there is still a long way to go to improve the cure rate of eBC.

Anesthesia is a fundamental technique for breast surgery, and it could be generally divided into two methods nowadays: inhalational general anesthesia (IGA) with anesthetic gases (majorly sevoflurane), and propofol-based anesthesia (PBA)^4^. The relationship between the anesthetic technique and breast cancer prognosis has not yet been clarified^5^. Surgical stress, opioids, and inhalation anesthesia have been reported to cause perioperative immunosuppression and thus increase cancer recurrence and decrease survival^6–9^. As the most widely used inhalation anesthetics, sevoflurane (SEVO) could suppress cell-mediated immunity (CMI) and promote tumor cell proliferation and angiogenesis through inducing the apoptosis of T lymphocytes and upregulating the expression of HIF-1a^10,11^. While propofol as an intravenous anesthetics does not suppress CMI, and instead it could increase cytotoxic T lymphocyte (CTL) activity, decrease inflammatory cytokine levels and suppresse COX-2 and PGE_2_ functions.Moreover, propofol could maintain the host innate immunity and immune defense via NK cells^11–18^, and thus propofol-based anesthesia has been supposed to increase the survival of cancer patients with surgery.

Although retrospective cohort studies have shown that the anesthetic technique may affect long-term survival after cancer surgery, whether PBA has an advantage over IGA in the prognosis of breast cancer patients is still controversial and inconclusive^19^. Several retrospective cohort studies have been published to address that PBA is generally associated with a better overall survival than IGA after breast cancer surgery. However, some recent retrospective studies concerning breast cancer showed that there were no differences between the two anesthesia methods in survival. Meanwhile, RCTs have not yet provided sufficient evidence that the anesthetic technique is associated with the recurrence rate or long-term outcomes in patients undergoing breast cancer surgery, possibly due to their limited sample size or short follow-up time^20–22^.

With increasing and conflicting results reported by different studies, we conducted the present meta-analysis to evaluate the controversial value on the survival of eBC for the two anesthetic techniques.

## Methods and materials

### Searching strategy and publication selection

We conducted a systemic literature search on16thJuly, 2022 of online databases, including EMbase, Pubmed, Scopus and Cochrane library (including Cochrane Central Register of Controlled Trials) for all studies concerning on propofol-based anesthesia and inhalational general anesthesia in early-stage BC patients by two reviewers (YJZ and PY) independently. The searching strategy isto search the following words in the title/abstract: (“breast cancer” OR breast) AND (“inhalation anaesthesia” OR isoflurane OR sevoflurane OR desflurane OR “nitrous oxide” OR “intravenous anaesthesia” OR “total intravenous anaesthesia” OR TIVA OR propofol). Only studies in English language were included.

Cross-referenced articles, and the potential relevant unpublished studies on important international conference websites, i.e. ESMO (European Society for Medical Oncology), ASCO (American Society of Clinical Oncology), and SABCS (San Antonio Breast Cancer Symposium), have also been gone through.

This study has been registered at PROSPERO2022 (ID:CRD42022334904. Available from: https://www.crd.york.ac.uk/prospero/display_record.php?ID=CRD42022334904).

### Inclusion and exclusion criteria

The inclusion criteria for eligible trials were as follows: (1) prospective/retrospective studies recruiting early-stage invasive BC patients (stage I–III); (2) patients should have undergone standard breast surgery, either mastectomy or breast conserving surgery under anesthesia; (3) studies should have included comparisons of long-term survival between IGA and PBA group; (4) detailed survival data, including overall survival (OS), recurrence-free survival (RFS), loco-regional recurrence-free survival (LRRFS) and distant metastasis-free survival (DMFS), etc., should have been presented in the studies. Definitions of survival data has been described in these studies; (4) In the case of studies derived from duplicate data sources, only the latest and the most comprehensive publication was included.

Studies meeting all the above criteria were included; otherwise, they were excluded.

### Data extraction and study objectives

Two independent investigators (YJZ and LB) performed the data extraction. The following information was extracted if available: author name, publication year, journal name, type of study, data source, enrolment period and clinical stage of patients, age range/median age, race, breast surgery type (mastectomy or breast conservation), number (No.) of patients in each group, detailed anesthetic techniques and drugs, median follow-up years, balanced or not in baseline characteristics of the two groups (with weighting methods). Survival data were extracted if available,otherwise, we extracted and transformed them from the survival curve. Hazard ratios (HRs) and the corresponding 95% confidence intervals (95% CIs) of three studies (RCTs as Yan 2018, Yan 2019 and Cho 2017) could also be found in the three previously published meta-analysis^21–23^.

The primary objective of the present meta-analysis was to evaluate the survival benefits of propofol-based anesthesia in eBC patients, compared with inhalational general anesthesia. The major index was OS, RFS, LRRFS and DMFS.

### Statistical analysis

HRs and the corresponding 95%CIs for PBA v.s. IGA on survival rate were extracted or calculated from each publication. Pooled HRs and 95% CIs for endpoints presented in forest plots were estimated using the Mantel–Haenszel method, using either fixed- or random-effects models. Statistical heterogeneity was evaluated using *I*^*2*^ statistics. When between-estimate heterogeneity was indicated (*I*^*2*^ > 25%), a random-effects model was applied.

The quality of the eligible cohort studies was assessed using the Newcastle–Ottawa Scale (NOS). The results demonstrated that the quality of all the included studies was generally high (score 8–9).

We also applied funnel plots and Egger’s test (indicated by p < 0.05) to determine the publication bias. All statistical analysis was performed with Stata 12.0 software. All tests were two-sided, and statistical significance was set at P < 0.05.

### Ethics approval and consent to participate

Ethics approval and trial registration numbers were not applicable to the present meta-analysis.

## Results

### Eligible studies

**Figure 1:**
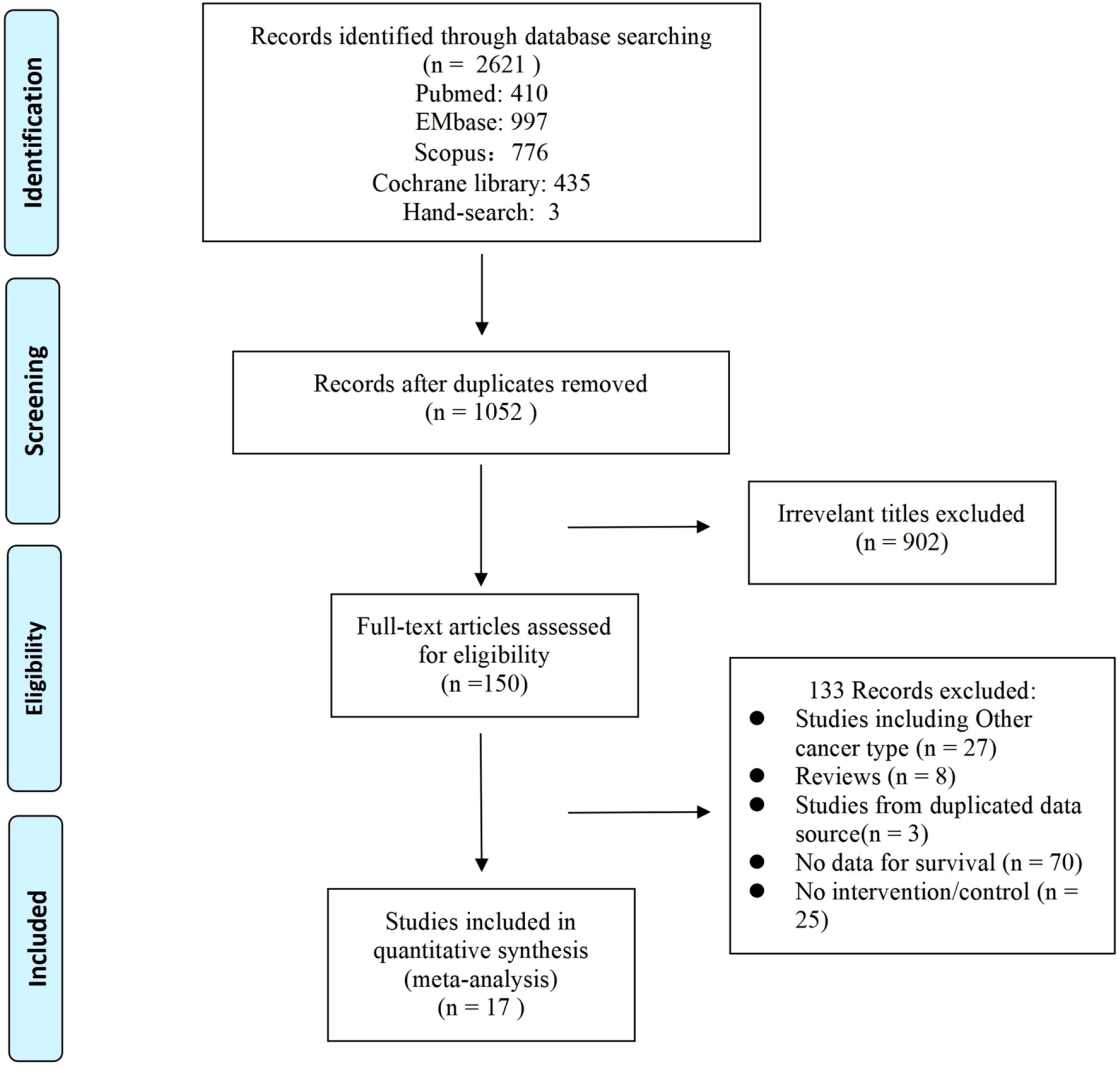
The PRISMA flow diagram.

A systematic search of databases and international conferences yielded two thousand six hundred and twenty-one (2621) publications. One thousand and fifty -two articles (1052) remained after excluding duplicates. After title and abstract screening, nine hundred and two (902) more articles were excluded. During the full-text review, a further one hundred and thirty-three (133) articles were excluded for the following reasons: Studies including Other cancer type (n = 27), Reviews (n = 8), Studies from duplicated data source (n = 3), No data for survival (n = 70), No intervention/control (n = 25). Ultimately, seventeen (17) publications meeting the inclusion criteria were recruited^24–37^. The PRISMA flow diagram is shown in Fig. 1.

### Characteristics of the enrolled studies

Among the seventeen eligible studies, thirteen^25–31,33,36–40^ were retrospective cohort studies, four^24,32,34,35^ were prospective randomized clinical trials (RCTs). All eligible studies published full-text articles. The characteristics and the corresponding extracted survival data of the eligible studies are presented in Tables 1–4, respectively.

**Table 1:** Basic information of eligible studies.

NO	Author	Year	Journal	Database	Country	Study type	Enroll-ment period	Median follow-up	Risk of bias assessment	Propofol usage	Volatile anesthetics
1	Enlund et.al. ^21^)	2020	ACTA ANAESTHESIOLOGICA SCANDINAVICA	Swedish Breast Cancer Quality Register	Sweden	Retrospective cohort	2006–2012	n.a	NOS score 8	Total intravenous anesthesia (TIVA)	Sevoflurane
2	Zhang et.al. ^34^)	2021	Am J Cancer Res	Taiwan Cancer Registry Database (TCRD)	China	Retrospective cohort	2009.1–2018.12	63.5m	NOS score 8	TIVA	Sevoflurane
3	Zhang et.al. ^35^)	2021	Biomedicine & Pharmacotherapy	Taiwan Cancer Registry Database (TCRD)	China	Retrospective cohort	2009.1–2018.12	62m	NOS score 8	PVB with propofol sedation	Sevoflurane
4	Zhang et.al. ^36^)	2022	Frontiers in Oncology	Health and Welfare Data Center (HWDC) established by Taiwan’s Ministry of Health and Welfare	China	Retrospective cohort	2009.1–2018.12	44-55m	NOS score 8	TIVA	Sevoflurane
5	Yan et.al. ^31^)	2018	BMC Anesthesiology	Cancer Hospital of Chinese Academy of Medical Sciences	China	Prospective RCT	2016.1–2016.8	25m	Low risk of bias	TIVA	Sevoflurane
6	Yoo et.al.^32^	2019	Anesthesiology	Seoul National University Hospital	Korea	Retrospective cohort	2005.1–2010.12	62m	NOS score 8	TIVA	Sevoflurane
7	Kim et.al.^26^	2017	Oncotarget	Severance Hospital, Yonsei University Health System	Korea	Retrospective cohort	2005.11–2010.12	70m	NOS score 8	TIVA	Sevoflurane
8	Lee et.al.^27^)	2016	Korean Journal of Anesthesiology	Korea Cancer Center Hospital	Korea	Retrospective cohort	2007.1–2008.12	ns	NOS score 8	TIVA	Sevoflurane
9	Enlund et.al.^22^	2014	Upsala Journal of Medical Sciences	Central Hospital in Västerås, Sweden	Sweden	Retrospective cohort	1998.1–2010.3	ns	NOS score 8	TIVA	Sevoflurane
10	Sessler et.al.^28^	2019	Lancet	Breast Cancer Recurrence Collaboration	Argentina, Austria, China, Germany, Ireland, New Zealand, Singapore and USA	Prospective RCT	2007.1–2018.1	36m	Low risk of bias	PVB with propofol sedation	Sevoflurane
11	Shiono et.al.^29^	2020	Journal of Anesthesia	Osaka University Graduate School of Medicine	Japan	Retrospective cohort	2008.1–2012.12	59m	NOS score 8	TIVA	Sevoflurane
12	Huang et.al.^25^	2019	PLOS ONE	Tri-Service General Hospital (TSGH), Taipei, Taiwan	China	Retrospective cohort	2006.1–2010.12	n.a	NOS score 8	TIVA	Desflurane
13	Yoon et.al.^33^	2022	Annals of Surgery	NHIS of the Republic of Korea	Korea	Retrospective cohort	2007–2016	42.1m	NOS score 8	TIVA	Sevoflurane
14	Yan et.al.^30^	2019	Cancer Management and Research	Cancer Hospital of Chinese Academy of Medical Sciences	China	Prospective RCT	2016.1–2017.1	25m	Low risk of bias	TIVA	Sevoflurane
15	Cho et.al.^20^	2017	International journal of medical science	Severance Hospital, Yonsei University Health System, Seoul	Korea	Prospective RCT	n.a	n.a	Low risk of bias	TIVA	Sevoflurane
16	Hong et.al. ^24^)	2019	BMC Anesthesiology	Chungnam National University Hospital	Korea	Retrospective cohort	2006.1–2009.12	n.a	NOS score 8	TIVA	Sevoflurane
17	Enlund et.al.^23^	2022	Anesthesiology	The Swedish PeriOperative Register and The National Quality Register for Breast Cancer	Sweden	Retrospective cohort	2013–2019	33m	NOS score 8	TIVA	Sevoflurane

*TIVA* total intravenous anesthesia, *PVB* paravertebral blocks, *RCT* randomized clinical trials, *NOS* Newcastle–Ottawa Scale, *n.a.* not available, *ref* References.

As shown in Table 1, two methods of propofol-based anesthesia (PBA) were reported in these publications: propofol-based total intravenous anesthesia (TIVA), and paravertebral blocks (PVB) with propofol sedation. For inhalational general anesthesia (IGA), most studies used sevoflurane as the inhalation anesthetics, while only one study used desflurane.

To note, Dr. Zhang et.al. published three articles and the patients were derived from Taiwan Cancer Registry Database (TCRD) during 2009/01/01 to 2018/12/31. However, the surgery method or PBA method for breast cancer patients were different in these three publications. Dr. Enlund et.al. also published three articles in Swedish population, but the inclusion period for patients and the derived database were not duplicated in the three articles. Thus we included all these publications in analysis.

As listed in Tables 2–4, HRs and their corresponding 95%CI for PBA versus IGA have been provided in most articles, while these information in three RCTs (Yan 2018, Yan 2019 and Cho 2017) with small sample size could be extracted from survival curve or from previous meta-analysis^20,21^.

**Table 2:** Sum-up of crude OS&RFS results in unweighted BC patients.

Author	Year	Journal	Breast cancer surgery	Sample Size		HRs and 95%CI for OS (PBA/IGA)	HRs and 95%CI for RFS (PBA/IGA)
				PBA	IGA		
Enlund et.al.^21^	2020	ACTA ANAESTHESIOLOGICA SCANDINAVICA	All	2967	3017	1-year 0.83 (0.75–0.93)	
Yan et.al.^31^	2018	BMC Anesthesiology	All	40	40	2-year 1.28 (0.94, 1.76)	2-year 0.89 [0.55, 1.42]
Kim et.al.^26^	2017	Oncotarget	All	56	1613	5-year 0.36 (0.09–1.50)	5-year 0.94 (0.41–2.16)
Lee et.al.^27^	2016	Korean Journal of Anesthesiology	Mastectomy	173	152	5-year 0.36 (0.02–8.58)	5-year 0.55 (0.31–0.97)
Enlund et.al.^22^	2014	Upsala Journal of Medical Sciences	All	620	1217	5-year 0.84 (0.65–1.08)	
Shiono et.al.^29^	2020	Journal of Anesthesia	All	212	814		1-year 0.86 (0.50–1.47)
Huang et.al.^25^	2019	PLOS ONE	All	344	632	5-year 1.17 (0.68–2.00)	
Yoon et.al.^33^	2022	Annals of Surgery	All	17,042	81,057	1-year 0.69 (0.55,0.88) 5-year 0.79 (0.70,0.88)	
Yan et.al.^30^	2019	Cancer Management and Research	All	42	38	2-year 0.84 (0.53, 1.31)	2-year 1.23 (0.67, 2.27)
Cho et.al.^20^	2017	International journal of medical science	All	25	25	2-year 1.14 (0.73, 1.79)	5-year 1.09 (0.77, 1.56)
Enlund et.al.^23^	2022	Anesthesiology	All	13,873	4.801	5-year 0.80 (0.70, 0.90)	

*HRs* Hazard ratios, *95% CIs* 95% confidence intervals, *IGA* inhalational general anesthesia, *PBA* propofol-based anesthesia, *OS* overall survival, *RFS* recurrence-free survival.

### Efficacy of propofol-based anesthesia

#### Crude overall survival in unweighted patients

**Figure 2:**
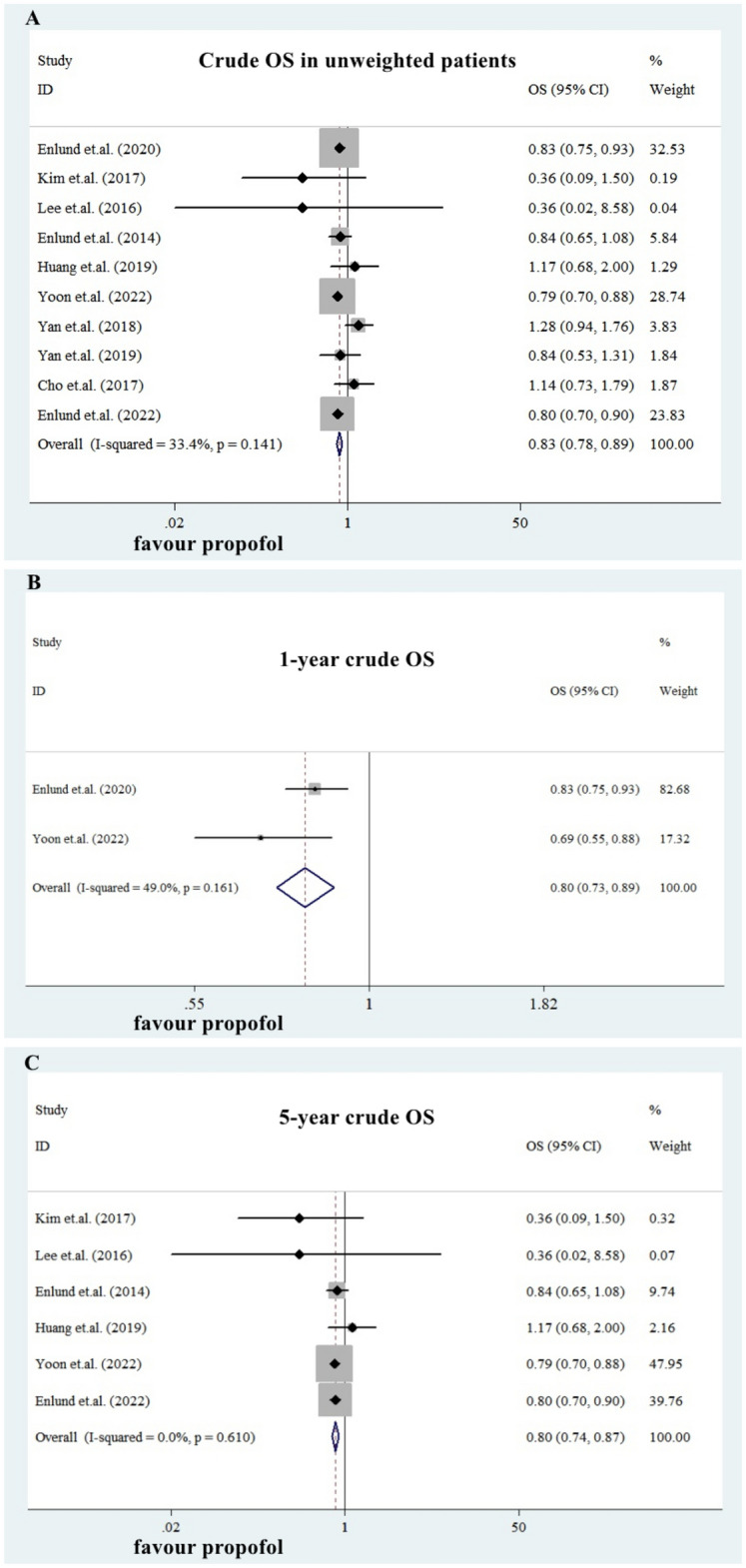
Efficacy of propofol-based anesthesia in unweighted patients. The summarized estimate HR of the PBA group versus the IGA group in unweighted patients (ten studies, N = 127,774, IGA group: 92,592, PBA group: 35,182.) was 0.83 (95% CI: 0.78-0.89. Figure 2A). Compared with IGA, PBA was associated with both better1-year OS (two studies, N = 104,083, IGA group: 84,074, PBA group: 20,009. PooledHR = 0.80, 0.73–0.89. Figure 2B) and 5-year OS (six studies, N = 121,580, IGA group: 89,472, PBA group: 32,108. HR = 0.80, 0.74-0.87. Figure 2C).

We treated the publications with unbalanced baseline characteristics (especially for T, N, M stage and ER, PR, HER-2 expressions) as unweighted eBC patients. The available overall survival (OS) data in such publications were curde OS. Ten studies provided data for crude OS in unweighted eBC patients (Table 2). Among them, three publications were prospective RCTs (Yan 2018, Yan 2019 and Cho 2017), but the recruited patients were unbalanced in baseline N stage and molecular subtypes. For crude OS, the separate and pooled HRs and 95% CIs were shown in Fig. 2A. No between-study heterogeneity was noted (p = 0.141, I-square = 33.4%). The summarized estimate HR of the PBA group versus the IGA group (ten studies, N = 127774, IGA group: 92592, PBA group: 35182.) was 0.83 (95% CI: 0.78–0.89).

However, the crude OS data provided in these ten publications had bias due to the different median follow-uptime: some were one-year OS data, while some were 5-year. To decrease the bias, we classified the data and conducted subgroup analysis. The summarized HRs for 1-year and 5-year OS were shown in Fig. 2B,C. Compared with IGA, PBA was associated with both better 1-year OS (two studies, N = 104083, IGA group: 84074, PBA group: 20009. Pooled HR = 0.80, 0.73–0.89) and 5-year OS (six studies, N = 121580, IGA group: 89472, PBA group: 32108. HR = 0.80, 0.74–0.87). The 2-year OS was similar in three publications but with a small sample size (three studies, N = 210, IGA group: 103, PBA group: 107. Pooled HR = 1.12, 95% CI: 0.90–1.40, forrest plot not shown).

### Overall survival in weighted patients

Although the above results demonstrated at the first time that PBA had a better OS than IGA in eBC patients, the limitation was obvious: the baseline characteristics in the two groups were extremely unbalanced. Thus, we could not apply the results in clinical practice directly. Fortunately, we figured out that many studies had also recognized it and used propensity score matching (PSM) method to decrease the bias. We treated these OS data derived from well-balanced patients after PSM as weighted OS.

**Figure 3:**
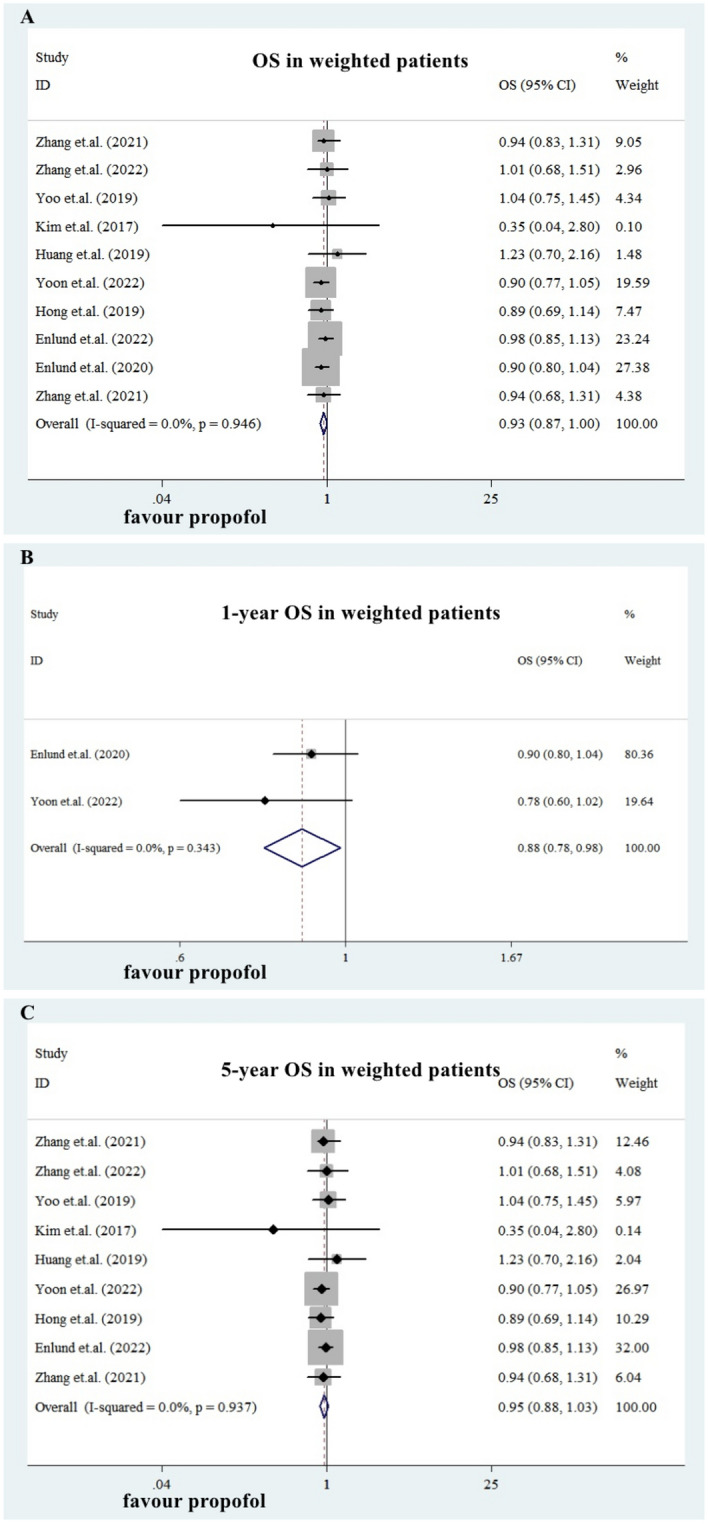
Efficacy of propofol-based anesthesia in weighted patients. The summarized estimate HR of the PBA group versus the IGA group in weighted patients (ten studies, N = 105,459, IGA group: 79,095, PBA group: 26,364.) was 0.93 (95% CI: 0.87-1.00. Figure 3A). Compared with IGA, PBA was associated with both better1-year OS (two studies, N = 83,007, IGA group: 67,609, PBA group: 15,398. PooledHR = 0.88, 0.78–0.98. Figure 3B) and a trend towards better 5-year OS (nine studies, N = 121,580, IGA group: 76,797, PBA group: 24,066. HR = 0.95, 0.88-1.03. Figure 3C).

Ten studies provided weighted OS data in these well-matched eBC patients (Table 3). They were all retrospective cohort studies. For these weighted OS, the separate and pooled HRs and 95%CIs were shown in Fig. 3A. No between-study heterogeneity was noted (p = 0.946, I-square = 0.00%). The summarized estimate HR of the PBA group versus IGA group (ten studies, N = 105459, IGA group: 79095, PBA group: 26364.) was 0.93 (95% CI: 0.87–1.00).

**Table 3:** Sum-up of weighted OS&RFS results in BC patients after PSM.

Author	Year	Journal	Breast cancer surgery	Sample Size		HRs and 95%CI for OS (PBA/IGA)	HRs and 95%CI for RFS (PBA/IGA)
				PBA	IGA		
Enlund et.al.^21^	2020	ACTA ANAESTHESIOLOGICA SCANDINAVICA	All	2298	2298	1-year 0.90 (0.80–1.04)	
Zhang et.al.^34^	2021	Am J Cancer Res	Breast conserving surgery (BCS)	1934	1934	5-year 0.94 (0.83–1.31)	
Zhang et.al.^35^	2021	Biomedicine & Pharmacotherapy	Breast conserving surgery (BCS)	1395	1395	5-year 0.94 (0.68–1.31)	
Zhang et.al.^36^	2022	Frontiers in Oncology	Mastectomy	707	707	5-year 1.01 (0.68–1.51)	
Yoo et.al.^32^	2019	Anesthesiology	All	1766	1766	5-year 1.04 (0.75–1.45)	5-year 1.04 (0.76–1.45)
Kim et.al.^26^	2017	Oncotarget	All	56	280	5-year 0.35 (0.04–2.80)	5-year 0.87 (0.29–2.62)
Sessler et.al.^28^	2019	Lancet	all	1043	1065		5-year 0.97 (0.74–1.28)
Shiono et.al.^29^	2020	Journal of Anesthesia	All	159	159		1-year 0.99 (0.45,2.19)
Huang et.al.^25^	2019	PLOS ONE	All	296	592	5-year 1.23 (0.70–2.16)	
Yoon et.al.^33^	2022	Annals of Surgery	All	13,100	65,311	1-year 0.78 (0.60,1.02) 5-year 0.90 (0.77,1.05)	
Hong et.al.^24^	2019	BMC Anesthesiology	All	154	154	5-year 0.89 (0.69, 1.14)	5-year 0.72 (0.41, 1.27)
Enlund et.al.^23^	2022	Anesthesiology	All	4658	4658	5-year 0.98 (0.85, 1.13)

We further classified the data according to the follow-up time and conducted subgroup analysis. The summarized HR for 1-year and 5-year OS was shown in Fig. 3B,C. Compared with IGA, PBA was associated with a better1-year OS (two studies, N = 83007, IGA group: 67609, PBA group: 15398. Pooled HR = 0.88, 0.78–0.98) and a trend towards better 5-year OS (nine studies, N = 121580, IGA group: 76797, PBA group: 24066. HR = 0.95, 0.88–1.03).

The summarized HRs for the weighted eBC patients indicated that PBA still had a significantly superior OS, when compared with IGA.

### Other survival data

We also conducted analysis in other survival data, including RFS, LRRFS and DMFS (Tables 3,4).

**Table 4:** Sum-up of weighted LRRFS&DMFS results in BC patients after PSM.

Author	Year	Journal	Breast cancer surgery	Sample Size		HRs and 95%CI for LRRFS (PBA/IGA)	HRs and 95%CI for DMFS (PBA/IGA)
				PBA	IGA		
Zhang et.al.^34^	2021	Am J Cancer Res	Breast conserving surgery (BCS)	1934	1934	5-year 0.77 (0.58–0.87)	5-year 0.91 (0.82–1.24)
Zhang et.al.^35^	2021	Biomedicine & Pharmacotherapy	Breast conserving surgery (BCS)	1395	1395	5-year 0.67 (0.46–0.99)	5-year 0.83 (0.63–1.10)
Zhang et.al.^36^	2022	Frontiers in Oncology	Mastectomy	707	707	5-year 0.52 (0.28–0.96)	5-year 0.74 (0.49–1.10)

**Figure 4:**
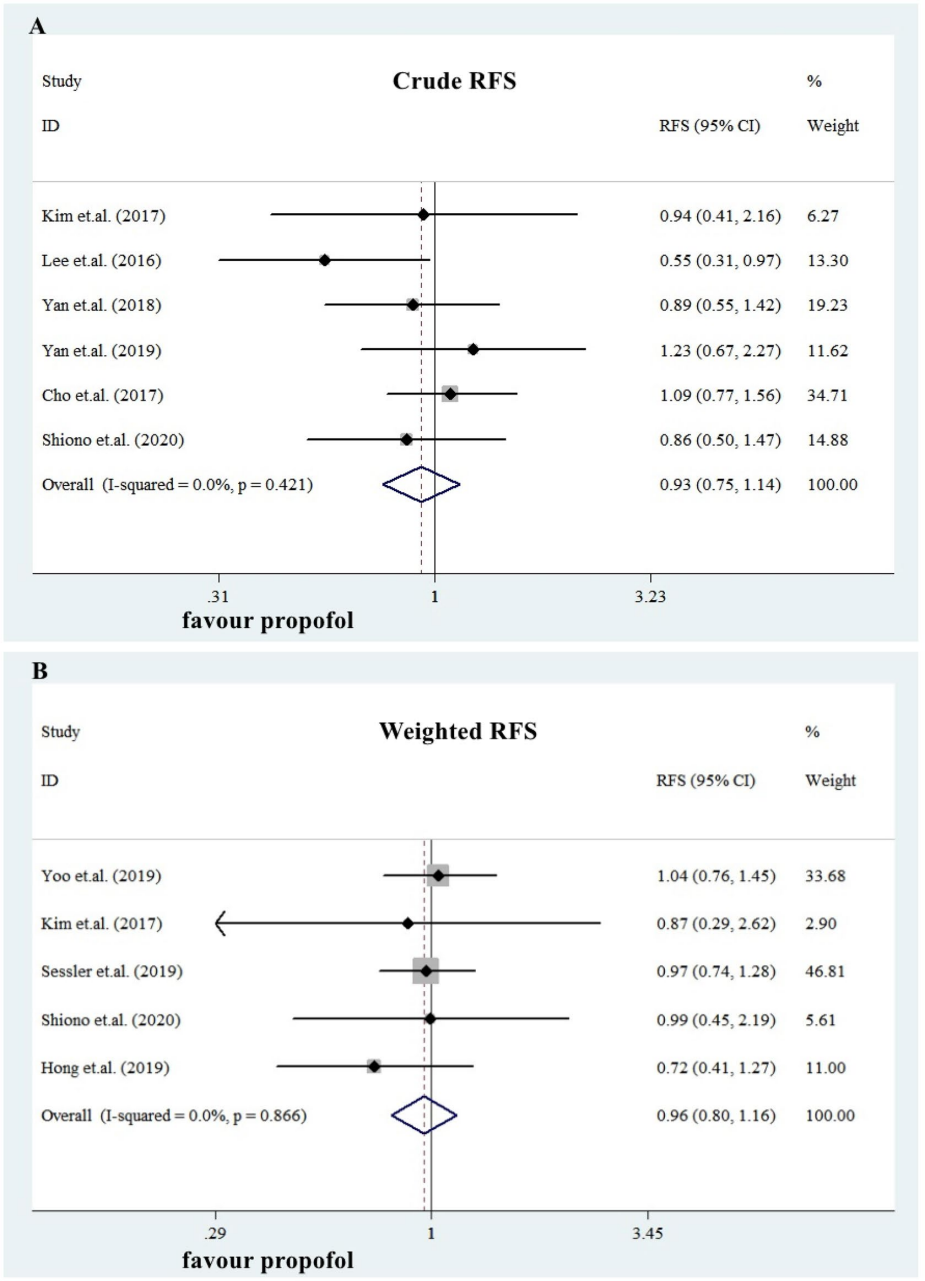
Impact of propofol-based anesthesia on RFS. For RFS, six studies provided crude data (N = 3230, IGA group: 2682, PBA group: 548.). The summarized HRs for crude RFS in PBA group was 0.93 (0.75, 1.14), see Fig. 4A. Five studies provided weighted RFS data after PSM in patients (N = 6602, IGA group: 3424, PBA group: 3178.). The summarized HRs for weighted RFS in PBA group was 0.96 (0.80, 1.16), seeFig. 4B.

For RFS, six studies provided crude data (N = 3230, IGA group: 2682, PBA group: 548.). The summarized HRs for crude RFS in PBA group was 0.93 (0.75, 1.14), see Fig. 4A. Five studies provided weighted RFS data after PSM in patients (N = 6602, IGA group: 3424, PBA group: 3178.). The summarized HRs for weighted RFS in PBA group was 0.96 (0.80, 1.16), see Fig. 4B.

**Figure 5:**
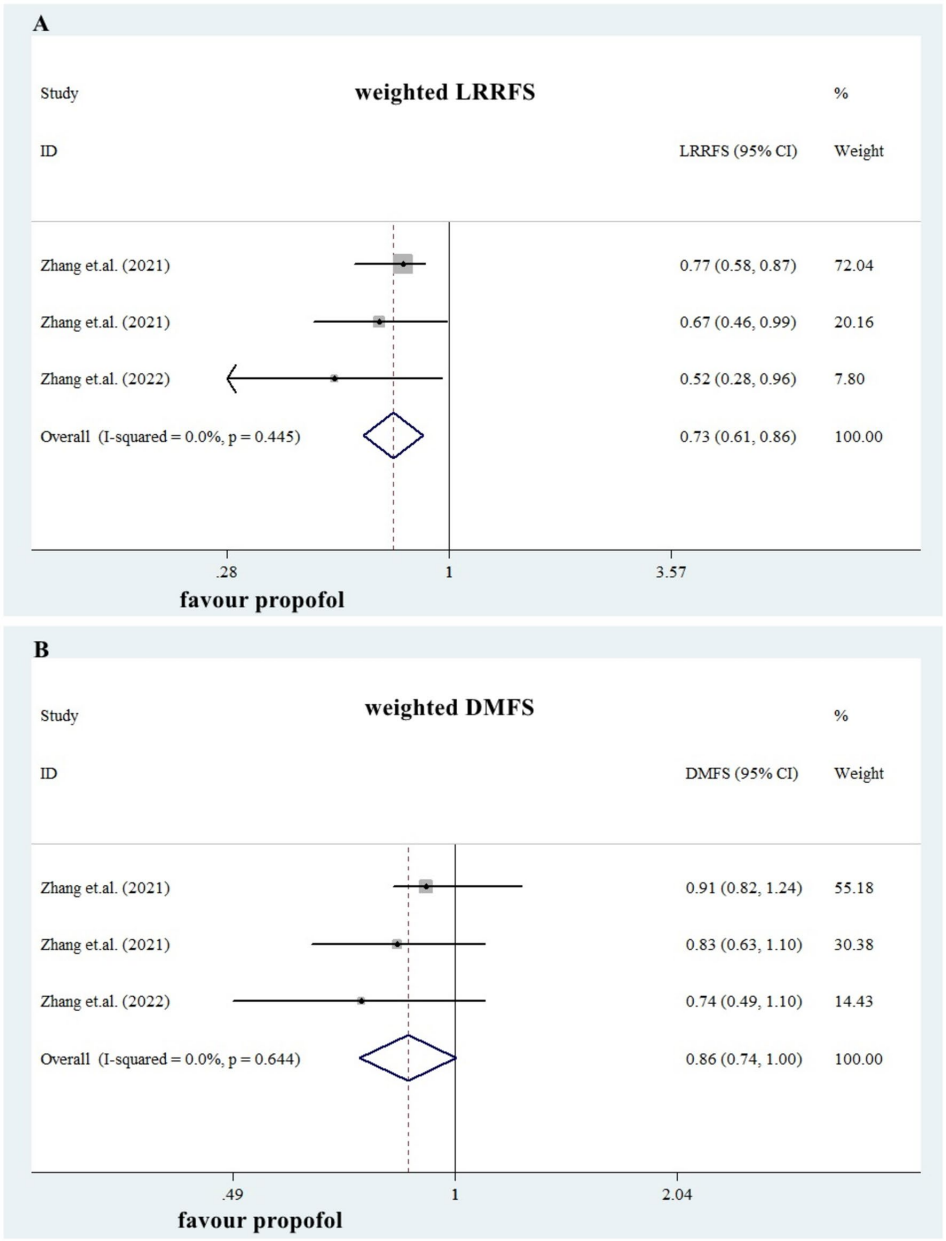
Impact of propofol-based anesthesia on LRRFS and DMFS. Three publications provided weighted LRRFS and DMFS data in well-balanced patients (N = 8072, IGA group: 4036, PBA group: 4036). The summarized HRs for LRRFS in PBA group was 0.73 (0.61, 0.86), while the summarized HRs for DMFS was 0.86 (0.74, 1.00), see Fig. 5A,B.

Three publications from Dr. Zhang et.al. provided weighted LRRFS and DMFS data (Table 4) in well-balanced patients (N = 8072, IGA group: 4036, PBA group: 4036) derived from Taiwan Cancer Registry Database, China. The summarized HRs for LRRFS in PBA group was 0.73 (0.61, 0.86), while the summarized HRs for DMFS was 0.86 (0.74, 1.00), see Fig. 5A,B.

### Publication bias

Egger’s test and funnel plots were used to detect and describe publication bias. No significant publication bias was identified in the data pooling (data not shown).

## Discussion

Whether the anesthesia technique and drugs influences the long-term outcomes of early stage breast cancer has been always an interesting question for oncologists and anesthesiologists. Propofol has been proved to be more favorable in breast cancer surgery than volatile general anesthetics (sevoflurane) in biomarker studies: a better inhibition on VEGF-C and TGF-β, more activated natural killer cells and higher cancer cell apoptosis.

However, the differences in long-term outcomes between propofol and volatile general anesthetics were not so apparent. The previous studies were few and they reported various survival results. The most important thing to note, most studies recruited limited and unbalanced patients with various follow-up time. Thus the bias in the these studies were inherent and concrete. Several meta-analysis have been published concentrating on this question, but due to the limited sample size and unweighted data, they were not convincing to end this argument.

For example, four published systemic reviews/meta-analysis concerning on our topic^19,21–23^. These four related previous reviews and meta-analysis have their disadvantages. Publications of Chang CY, et.al. and Yap A, et.al. included several kinds of cancers and did not concentrate on anesthetic technique and and prognosis breast cancer. Thus the number of eligible studies were very limited (4 included studies). Publication of Pang QY, et.al.concentrate on the side effects of anesthetic technique, thus the number of eligible studies on survival was also very limited (3 included studies). Only Lv R, et.al. include 7 studies. Moreover, the most important issue of all the four studies is, the baseline characteristics in the include studies are unbalanced, which brings the major bias.

In the present meta-analysis, we included many latest studies with relative large sample sizes (17 studies). More importantly, many studies applied propensity score matching methods (PSM) to balance the baseline characteristics between propofol group and inhalation anesthetics group. PSM is a method to minimize selection bias between interventional groups when estimating causal intervention effects in uneven population. The treatment groups (propofol- or inhalation- anesthesia) were matched on a propensity score. The propensity score is the probability of intervention assignment conditional on the current baseline characteristics. For breast cancer patients, we treated the TNM stage and molecule subtypes (ER, PR, HER-2 expressions) as the most important characteristics. Patients should be balanced in these index after PSM. Further, we have conducted analysis according to the crude or weighted data. Thus the bias in our study has been kept to an absolute minimum, when compared with previous studies.

The most important message of present meta-analysis is that propofol-based anesthesia is associated with a better overall survival in early-stage breast cancer patients, when compared with inhalation-maintained anesthesia. It has been a long-expected but unproven clinical answer so far. The benefits could be found in both unweighted and weighted patients. Thus we think that these results could bring important impacts to the anesthesia choice for breast cancer surgery: propofol-based anesthesia should be preferred over inhalation anesthesia. We are also looking forward to the results of more well-designed undergoing RCTs, including the “CAN Study”^41^, to further confirm this question.

However, we should recognize that anesthesia period during surgery is short and once-only use for most breast cancer patients, and thus its function is hard to present. We also acknowledge that most anesthesia today are mixed, with both propofol and volatile anesthetics applied. In our cancer center, propofol are popularly used in the anesthesia induction, while volatile anesthetics are used for anesthesia maintaining. Thus, a non-propofol or a non-inhalation anesthesia are not popular nowadays, which could bring difficulties in analyze the clinical outcomes between propofol and volatile anesthetics.

Another important influencing factor is the status of eBC patients receiving surgery: whether they are newly diagnosed, or treated after neoadjuvant therapies (chemotherapy, endocrine therapy, anti-HER2 therapy, radiotherapy). In theory, the benefits of propofol mainly contribute to its function on the immune system and disseminated tumor cells during surgery. Then its efficacy may be greatly weakened in those patients with a complete or good partial remission after neoadjuvant therapies. In recent years, the ratio of neoadjuvant therapies has been increased significantly in eBC patients, which would also influence whether the patients benefit from the anesthesia technology during surgery.

After all, if we decided to apply the propofol-based anesthesia, then the next question is which is the optimal technique: propofol-based total intravenous anesthesia or paravertebral blocks with propofol sedation. In the two technology, the propofol dose and drug concentration in blood are different. More clinical trials could be conducted to further elucidate which propofol-based anesthesia could be better.

## Conclusions

The present study is the first comprehensive meta-analysis to demonstrate that propofol-based anesthesia could significantly improve OS and LRRFS in non-metastatic breast cancer patients, compared with inhalational anesthesia. More prospective RCTs were needed to further clarify the target population of PBA and the optimal anesthesia usage for propofol.

## Data Availability

The data used to support the findings of this study are included within the article.
